# Cost and feasibility: an exploratory case study comparing use of a literature review method with questionnaires, interviews and focus groups to identify barriers for a behaviour–change intervention

**DOI:** 10.1186/s12913-015-0877-1

**Published:** 2015-05-29

**Authors:** Andria Hanbury, Katherine Farley, Carl Thompson

**Affiliations:** Department of Health Sciences, University of York, York, UK; Faculty of Medicine and Health, University of Leeds, Leeds, UK

**Keywords:** Barriers, Diagnostic analysis, Costs, Feasibility, Tailored intervention, Implementation

## Abstract

**Background:**

It is often recommended that behaviour-change interventions be tailored to barriers. There is a scarcity of research into the best method of barrier identification, although combining methods has been suggested to be beneficial. This paper compares the feasibility and costs of three different methods of barrier identification used in three implementation projects conducted in primary care.

**Methods:**

Underpinned by a theory-base, project one used a questionnaire and interviews; project two used a single focus group and questionnaire, and project three used a literature review of published barriers. The feasibility of each project, as experienced by the research team, and labour costs are summarised.

**Results:**

The literature review of published barriers was the least costly and most feasible method, being quick to conduct and avoiding the challenges of recruitment experienced when using interviews or a questionnaire. The feasibility of using questionnaires was further reduced by the time taken to develop the instruments. Conducting a single focus group was also found to be a more feasible method, taking less time than interviews to collect and analyse the barriers.

**Conclusions:**

Considering the ease of recruitment, time required and cost of the different methods to collect barriers is crucial at the start of implementation studies. The literature review method is the least costly and most feasible method. Use of a single focus group was found to be more feasible than conducting individual interviews or administering a questionnaire, with less recruitment challenges experienced, and quicker data collection. Future research would benefit from comparing the robustness of the methods in terms of the comprehensiveness of barriers identified.

**Electronic supplementary material:**

The online version of this article (doi:10.1186/s12913-015-0877-1) contains supplementary material, which is available to authorized users.

## Background

At the outset of implementation projects aimed at improving clinical practice, it is recommended that the intervention needs to be tailored to barriers of the targeted behaviour [1]. However, there is a dearth of guidance available for researchers weighing up the pros, cons and resource implications of the different methods of barrier identification. There is no “standard” approach to barrier identification [2], with researchers often opting to combine methods. Commonly-used methods include questionnaires, qualitative interviews and focus groups. Literature reviews can also to identify barriers, an approach advocated in the intervention mapping literature [3]. Methods used are often not justified in publications, or justified on the merits of the method in general (e.g., qualitative interviews to provide contextual richness). The merits of the method in relation to barrier identification (e.g., qualitative methods due to anticipated response rate difficulties with use of a questionnaire) are not commonly discussed. In response to the scarcity of research into the optimal methods of barrier identification, Baker et al. [4] in a Cochrane review of tailored interventions for overcoming barriers to change called for more research into this area.

The lack of attention afforded to barrier identification is surprising given its importance when developing interventions: if important barriers are not identified, they cannot be targeted, limiting intervention effectiveness. However, one recently published international study [5] has systematically compared the feasibility of five different methods of identifying barriers to health professionals’ adoption of recommendations for the management of different long term conditions. Involving five European countries, with one research team in each country targeting a different set of recommendations, the methods of brain storming by health professionals, interviews with health professionals, and interviews with patients were directly compared for the number of barriers and unique barriers relating to the uptake of recommendations identified. The added value of conducting structured group discussions (guided by a checklist) after brainstorming, and using questionnaires were also assessed. Feasibility of the methods was evaluated through examination of researchers’ diaries, where they noted any problems experienced and time required and through interviews with one researcher from each team. Of all of the methods, questionnaires were found to be most problematic, and the in-depth data gained from interviews, whilst regarded positively by users, incurred higher costs due to the time required for recruiting to and analysing interview data. Importantly, it was found that the different techniques identified different barriers. The authors recommend, therefore, using a mix of techniques, with braining storming supplemented with structured group discussion (rather than individual interviews) a simple and minimal cost methodology.

The cost of different methods is an important consideration. Different methods make different resource demands, largely due to the time required to carry them out. The overall cost effectiveness of changing health professionals’ behaviour has started to receive attention [6-8], although reporting of this information is currently rare [9]. To determine cost effectiveness, Mason proposes the ‘Policy Cost Effectiveness’ framework, requiring calculation of the total costs of the project, the additional number of patients who receive the targeted treatment as a consequence of the project, and the per patient cost effectiveness of the recommended treatment targeted by the project. Project costs largely come from the labour (for example, costs of conducting barrier identification work) and associated salary oncosts (e.g., national insurance, pension contributions), as well as additional costs incurred during intervention development and delivery, such as printing of intervention materials and travel. Costs are particularly pertinent for implementation studies because the interventions have to compete with other quality improvement activities in primary and secondary care settings with limited budgets. Therefore, the labour cost of diagnosing barriers to health professionals’ adoption of recommendations in particular requires consideration, alongside the feasibility of methods (recruitment of health professionals and time taken to identify barriers). Using potentially resource-intensive methods, particularly when combining methods, has implications for project costs, and subsequently the overall cost effectiveness of an intervention.

This paper outlines three implementation studies conducted in a primary care setting as part of a larger research programme [10] conducted between 2009 and 2013. Three different methods of barrier identification were used in combination across two of the studies: theory-based questionnaire (using electronic and paper-based delivery) and interviews (project one), and a theory-based questionnaire (electronic delivery only) and focus group (project two). For project three, a brief desk-top based literature review of published barriers was used, followed by checking the applicability of the barriers locally with a small sample of health professionals. The labour and associated costs, and feasibility of the different methods as experienced by the research team, are compared to answer the research question: ‘which is the most feasible method of identifying barriers to changing clinical practice?’ Suggestions for future studies based on these findings are made.

## Methods

Ethical approval was granted for this study by National Research Ethics Service (Reference 10/H1311/1). The research programme was a series of implementation-themed research studies aimed at improving primary care health professionals’ uptake of research-based recommendations into practice across 83 general practices within one regional health economy in Northern England. The three projects that form the focus of this exploratory case study paper are summarised in Table 1.

**Table 1:** Summary of projects 1–3

Project	Aim and targeted health professionals	Method/s of barrier identification
One	To increase GPs, health visitors’ and nurse practitioners’ referrals for women diagnosed with mild to moderate postnatal depression to psychological therapies, recommended by National Institute for Health and Clinical Excellence (NICE).	A questionnaire measuring constructs from Greenhalgh et al’s conceptual model of the determinants of diffusion [17], followed by qualitative interviews with seven local health professionals.
Two	To increase GPs and nurse practitioners’ referral of symptomatic patients for spirometry testing to confirm diagnosis of chronic obstructive pulmonary disease, recommended by NICE.	Single focus group with health professionals and practice managers (10 general practitioners, 3 practice managers and 3 nurses), with questions guided by constructs from the theory of planned behaviour (TPB) [15]. This was followed by a TPB questionnaire, the content of which was based upon barriers identified from the focus group.
Three	To increase GPs and nurse practitioners’ opportunistic screening of patients for alcohol misuse using a validated screening tool, recommended by NICE.	Literature review to identify barriers to screening for alcohol misuse in primary care. Barriers were organised into thematic groupings using the theoretical domains framework [13], and checked for their applicability in the local context with an opportunistic sample of health professionals (11 general practitioners and 1 nurse). This was done using a checklist summarising each of the barriers and asking the health professionals to indicate whether they considered them to apply locally. The barriers were also discussed for their relevance and amenability to change with the project stakeholder group, comprising researchers, health professionals, and members of the local, collaborating, quality improvement team.

### Calculation of labour and associated costs

Each project involved NHS quality improvement colleagues due to the overall research programme being a theme from the Leeds, York and Bradford Collaboration for Leadership in Applied Health Research and Care (CLAHRC). The CLAHRCs promote collaborative working and knowledge transfer between academia and the NHS, and this is reflected in the size of the team, although a majority of the team were not funded full time to work on the overall research programme. The staff on the team also worked on other projects and so contributors provided their best estimates of labour contributions to the identification of barriers work for each project, and the identification of the clinical recommendation to target. This activity was the same across all three projects (consultation with local stakeholders), except that for project one, topic selection also employed a more detailed methodology which required an additional survey to be administered compared with projects two and three. Therefore, the costs of project one are inflated by comparison. Time estimations were made retrospectively by team members on completion of each project, rather than through use of time sheets; calendar templates were provided to help with estimation and took into account contributor’s working hours. For ‘major contributors’ (mostly research team members), the fraction of labour dedicated to each project on a monthly basis was reported. For ‘minor contributors’, estimations of labour contributions were made using smaller labour units (e.g. weeks/days/h).

### Labour costs

The full economic cost of a unit of a team members’ labour was estimated based on the methodology used in the Unit Costs of Social and Health Care [11]. Midpoints of team members’ salary pay bands were combined with estimates of salary oncosts (Employers’ NI and pension contributions) and overhead and capital cost estimates. This allowed estimation of the full annual economic cost of the contributors’ labour. Overhead and capital cost figures were taken from estimates provided in the Unit Costs of Social and Health Care [11] for Scientific and Professional Staff. Full annual cost estimates were broken into appropriate units of labour (month/week/day/h). The economic cost of each individual’s contribution was estimated by multiplying the estimated number of labour units (weeks/days/h) by the appropriate estimate of the full economic cost per unit.

### Direct costs

The costs of printing and posting paper-copies of questionnaires (for project one) were included in the costing, identified from invoices/bills for project one. Project two used only electronic administration of the questionnaire.

### Feasibility

Assessment of the feasibility of each method was based on consideration of the ease of recruitment of health professionals to conduct barrier identification work and time taken to identify barriers. Any additional challenges experienced were also noted, discussed at monthly ‘operational group meetings’ attended by academic and NHS team members.

## Results

Table 2 summarises the labour costs, alongside the research teams’ experience of the ease of recruiting health professionals to conduct the barrier identification work and the time taken to identify the barriers. Additional files 1, 2, and 3 summarise the calculation of labour and associated costs for each project.

**Table 2:** Summary of cost and feasibility of barrier identification work for projects 1–3

Project	Cost of barrier diagnosis work (see Additional files 1, 2, and 3 for cost calculations and contribution of team members)	Feasibility
1	Total cost £228,609.12	Questionnaire
	(labour = £224,256.68; direct costs = £4352.44)	•Required several iterations to develop, including piloting with sample of health professionals based at one general practice in a different geographical region, arranged through contacts within the research team (survey was sent electronically to 29 general practitioners, of which 12 responded with feedback) .
		•Recruitment was difficult, with a low response rate (19 %), despite providing 2 reminders and a paper copy of the questionnaire to non-responders
		•Developing, piloting, revising and administering the questionnaire and reminders, and the subsequent analysis of the data took approximately 8 months, with questionnaire drafting started in March 2010, and the data analysed by October 2010.
		Interviews
		•Recruitment was difficult: of an initial random sample of 20 health professionals sent a letter of recruitment, no responses were received, despite offering a £20 gift voucher as an incentive for taking part and the quality improvement team telephoning those who had received a letter. Gatekeepers (general practice managers) were subsequently used to recruit health professionals (the quality improvement team worked through the list of practices, talking to practice managers to gate-keep and encourage willing GPS to participate).
		•To develop the interview schedule, send letters of invitation, conduct follow-up phone calls, contact practice managers, conduct and then transcribe and analyse 7 interviews took approximately 5 months, with the process beginning in July 2010 and being completed in November 2010.
		The questionnaire and interview methods combined, therefore, took approximately 9 months, starting in March 2010 and concluding in November 2010.
2	£59,834.31	Questionnaire
	(labour = £59,834.30; direct costs = £0)	•This used a modified version of the questionnaire used for project one. The questionnaire was piloted with 5 health professionals based at different practices within the collaborating primary care trust who were known to have an interest in the clinical topic by the quality improvement team. Questionnaire was only sent electronically
		•Recruitment was challenging: low response rate (15 %), despite sending 2 reminders
		•To modify the questionnaire, pilot, revise, administer, send the reminders, and the subsequent analysis of the data collected took approximately 4 months, with work beginning on the questionnaire in February 2012, and analysis being completed in May 2012.
		Focus group
		•Easy to arrange due to opportunity to attend a pre-arranged meeting to collect barriers, facilitated by quality improvement team colleagues. The meeting was attended by 10 general practitioners, 3 practice managers and 3 practice nurses.
		•Arranging to attend and then attending single meeting for data collection, developing the focus group questions, transcribing and analysing the data took approximately 3 months, with initial email enquiry to NHS colleagues regarding potential to attend meeting sent November 2011, and the focus group held and the barriers collected and analysed in January 2012.
		The questionnaire and focus group method combined, therefore, took approximately 7 months, starting in November 2011 and concluding in May 2012
3	£34,725.65	Published barriers
	(labour = £34,725.65; direct costs = £0)	•No challenges encountered
		•To develop literature search strategy, run the search, read articles and summarise the barriers identified in them, discuss barriers as a team (to check coding of them), share with a sample of health professionals (11 general practitioners and 1 practice nurse) to check local relevance and develop a logic model took approximately 2 months, with the process starting in August 2012 and being completed by October 2012

*Labour costs are inflated for this project due to use of a questionnaire to guide topic selection compared with projects 2 and 3 which used only consultation with stakeholders to guide topic selection

For project one, using a questionnaire followed by seven individual interviews, it took approximately nine months to complete the barrier identification work. Of this, eight months were spent developing, delivering and analysing the questionnaire data, and five months on interviews, with the interviews conducted at the same time that the questionnaire data was analysed due to the interviews being conducted after the questionnaire. For project two, the questionnaire was less time consuming to develop (four months) and due to only being delivered electronically, did not incur ‘direct project costs’ of printing and delivery (for project one this cost an additional £4352.44). Combining the questionnaire with a single focus group was found to be less time consuming than combining it with individual interviews as in project one, but still took seven months overall, of which the questionnaire took four months and the focus group took three months. It was not possible for the focus group work to overlap with the questionnaire work, as in project one, due to the focus group work helping to inform questionnaire design/content. For project three, the literature review of published barriers was specifically selected as a brief method for comparison with the combined methods. It was found to be a feasible method of barrier identification: quick to conduct (two months from start to finish), with no challenges experienced.

Considered on their own, the feasibility of using questionnaires or interviews was reduced through the difficulty experienced in recruiting health professionals, with the questionnaire achieving response rates of 15 and 19 % for projects one and two respectively, and recruitment to the interviews requiring us to go through practice managers as ‘gate keepers’ after failing to recruit via letters of invitation. This made the collection of barriers more time consuming. As projects one and two used combined methods, the costs of the barrier identification work was further increased and the feasibility further reduced. Reflecting the increased labour required to collect barriers using combined methods, the barrier identification work for project one cost £228,609.12 and for project two it cost £59, 834.31. For project three, the cost was £34,725.65. Whilst the costs of project one are inflated due to the development and administration of an additional questionnaire during the process of topic selection, the cost of project two is still substantially higher than project three.

## Discussion

This paper has compared the labour costs and the feasibility of different methods of barrier identification used within three theory-based implementation projects to identify the most feasible method/s.

Conducting a literature review to identify barriers from published papers was found to be more feasible than use of a questionnaire, interviews or focus group. The literature review method removed the challenge of recruiting busy health professionals, which was experienced in project one (which used a questionnaire and interviews) and project two (which used a questionnaire and focus group). Conducting the literature review was, therefore, quicker than the other methods (for example, taking two months compared with five months for interviews, or three months for the focus group). The literature review method was used partly to reduce the resources required but also to experiment with a briefer form of barrier identification. Importantly, our NHS partners’ desired to speed up the process of barrier identification, with pressure to be seen to be intervening quickly to address low adoption rates once they had been identified. However, whilst this method was found to be the most feasible, it is acknowledged that for some clinical topics, identifying published barriers papers could be more difficult than it was in this particular study, with either a high volume of papers (which would be more time consuming, and, therefore, more costly in terms of labour time), or very few papers available.

Using combined methods for barrier identification is recommended, increasing robustness through comparing barriers collected from multiple sources [12]. However, this study drawn attention to the potential for increased costs when combining methods; for example, with project two costing £59, 834.31, compared with £34,725.65 for project three which used a single method. When considering combining methods, it is, therefore, particularly important to consider the cost and feasibility of the methods to be combined, as well as the order in which the methods will be used. For example, if qualitative methods are to be used to identify barriers to then inform the design of a questionnaire, the methods cannot be run concurrently, compared with if the qualitative methods are to be used purely to enable a richer exploration of barriers alongside use of a questionnaire.

Questionnaires were found to be the least feasible method. They were time consuming to develop (requiring multiple iterations and piloting to ensure optimal design), and despite this effort, response rates were low (15–19 %). Project costs were then increased through also providing paper copies of the electronic questionnaire in project one (incurring costs of printing and administration: an additional £4352.44 in project one), and feasibility reduced through having to use multiple reminders to try and bolster response rates, which introduced time delays. Similar challenges to the use of questionnaires were also reported by Kraus et al. [5] in their assessment of the feasibility of different methods of barrier identification. For project one, the questionnaire method took approximately eight months, and in project two it took approximately four months. Comparing projects one and two, the feasibility of questionnaires may be increased through opting for only electronic administration to keep costs lower, and through modifying ‘off the shelf’ questionnaires where they are available. For example, if using the theoretical domains framework [13] to underpin barrier identification, a survey has been developed that would require minimal input to adapt it to different settings [14], and if using the theory of planned behaviour [15] (as in project two), questionnaire development is facilitated through making use of guidance on to how to operationalise the constructs [16]. However, for use in the NHS on a rolling basis across a number of implementation studies, the need to consistently achieve satisfactory response rates is unlikely to be feasible. Further challenges with the use of questionnaires are also likely to arise once the data is collected, with low response rates limiting the robustness and generalizability of the findings.

Qualitative methods are recommended in the early stages of intervention development in the revised Medical Research Council Framework for the development and evaluation of complex interventions [1]. Comparing projects one and two which used qualitative methods alongside a questionnaire, interviews were found to be a less feasible qualitative method of barrier identification than use of a single focus group, with the interviews taking five months from start to finish, compared with the focus group which took three months. This was largely due to the challenge experienced in recruiting health professionals to take part in interviews. No responses were received to the initial round of interview recruitment letters sent to a randomly drawn sample. Subsequently, we had to go through practice managers who acted as gatekeepers to recruitment, which caused time delays. Time and, consequently, labour costs were further increased through having to conduct, transcribe and analyse individual interview data which took longer for the interviews than the focus group. For example, it was necessary to conduct each of the seven interviews on different days and in different locations to facilitate recruitment. The focus group, by comparison collected the views of 10 general practitioners, three practice nurses and three practice managers in a single meeting. However, the feasibility of this method was increased by the opportunity to conduct the focus group during a pre-arranged meeting which was brought to our attention via the collaborating quality improvement team. Without this opportunity, it is likely that the same recruitment challenges experienced for the interviews would have occurred, with the added complexity of trying to coordinate busy health professionals’ diaries. This highlights the importance of having well established collaborations in place with local quality improvement colleagues in the collaborating organisation: these team members were able to use existing links with practice managers to facilitate recruitment for the interviews, and to advise regarding the opportunity to conduct the focus group at a pre-arranged meeting.

This study has several limitations. The method used to estimate time spent on each project was based on retrospective self-reports by each team member. For each project, estimation was provided on completion of the project (on average, completion being approximately 12 months after the period of barrier identification) and, therefore, will be subject to some unreliability, despite team members being prompted to use calendar templates to facilitate recall and reduce the risk of recall bias. Additionally, due to the overarching research programme being organised into phases: a ‘developmental phase (topic selection and barrier identification), followed by an implementation and evaluation phase, costs were estimated for topic selection work with stakeholders and barrier identification work combined, rather than per method used. This limits our ability to make direct comparisons of the costs of the different methods, especially since both projects one and two used combined methods. In basing the comparison of barrier identification methods on those employed across three studies within an overarching research programme, the full array of different methods and combination of methods had not been attempted, nor was possible. The final limitation of this study is the absence of an assessment of the ‘comprehensiveness’ or robustness of the barriers identified (whether all important barriers have been identified); consideration of this would also have enabled evaluation of the costs in relation to effectiveness of the methods employed. However, there is no established standard for assessing this; in their review of barrier identification methods, Krause et al. counted the number and type of barriers identified using each method. The number of barriers is, however, only a rough yardstick measure, and the more barriers identified, the greater the need for, and the greater the challenge becomes of tailoring the intervention to those most important barriers. Each project used a different theory-based to underpin the exploration of barriers, with the identified barriers coded against the theoretical constructs in the subsequent phase of the research programme. However, whilst this may enable a rough gauge as to the variety of barriers identified, it gives little confidence as to whether the most influential barriers have actually been identified. Using qualitative methods in projects one and two alongside the questionnaire, and engagement with stakeholders in project three, however, provided some assurance that the main barriers had been identified using each method/combination of methods.

## Conclusions

This paper highlights important factors for consideration by researchers at the start of a theory-based implementation project, given the importance of the barrier identification work in influencing project timescales, project costs, and eventually influencing intervention design through the barriers identified. Essentially, the feasibility (time and ease of collecting barriers) and relative labour costs should be considered at the outset of such projects, given the context of competing priorities for the spenditure of tight budgets in the health services. The findings of this study suggest that there may be merit in using the less resource intensive literature review method, or a focus group, rather than use of more resource intensive interviews or questionnaires which appear to be less feasible. It is recommended that the former be accompanied by a review of the identified barriers by local health professionals, and the latter facilitated through opportunity to conduct the focus group at a pre-arranged meeting or workshop. Future research would benefit from comparing the brainstorming with structured group discussion method [6], with the literature review method identified here as most feasible, as well as comparing the robustness of the methods in terms of the comprehensiveness of barriers identified.

## Additional files

Additional file 1:
**Cost Estimation.** Project one. Additional file 2:
**Cost Estimation.** Project two. Additional file 3:
**Cost Estimation.** Project three.
